# Genotype-specific interactions and the trade-off between host and parasite fitness

**DOI:** 10.1186/1471-2148-7-189

**Published:** 2007-10-05

**Authors:** Lucie Salvaudon, Virginie Héraudet, Jacqui A Shykoff

**Affiliations:** 1 Laboratoire Ecologie, Systématique et Evolution, UMR 8079, Univ Paris-Sud, Orsay cedex, F-91405; CNRS, Orsay cedex, F-91405; AgroParisTech, Orsay cedex, F-91405 France

## Abstract

**Background:**

Evolution of parasite traits is inextricably linked to their hosts. For instance one common definition of parasite virulence is the reduction in host fitness due to infection. Thus, traits of infection must be viewed in both protagonists and may be under shared genetic and physiological control. We investigated these questions on the oomycete *Hyaloperonospora arabidopsis *(= *parasitica*), a natural pathogen of the Brassicaceae *Arabidopsis thaliana*.

**Results:**

We performed a controlled cross inoculation experiment confronting six lines of the host plant with seven strains of the parasite in order to evaluate genetic variation for phenotypic traits of infection among hosts, parasites, and distinct combinations. Parasite infection intensity and transmission were highly variable among parasite strains and host lines but depended also on the interaction between particular genotypes of the protagonists, and genetic variation for the infection phenotype of parasites from natural populations was found even at a small spatial scale within population. Furthermore, increased parasite fitness led to a significant decrease in host fitness only on a single host line (Gb), although a trade-off between these two traits was expected because host and parasite share the same resource pool for their respective reproduction. We propose that different levels of compatibility dependent on genotype by genotype interactions might lead to different amounts of resources available for host and parasite reproduction. This variation in compatibility could thus mask the expected negative relationship between host and parasite fitness, as the total resource pool would not be constant.

**Conclusion:**

These results highlight the importance of host variation in the determination of parasite fitness traits. This kind of interaction may in turn decouple the relationship between parasite transmission and its negative effect on host fitness, altering theoretical predictions of parasite evolution.

## Background

Understanding the selective forces driving parasite evolution is crucial in the fight against infectious diseases, both in agriculture and human health. Darwinian medicine aims at controlling the evolution of human pathogens in order to drive them toward "milder" forms or, ideally, to extinction [1]. Such control requires knowledge of which traits of infections are adaptive for the host or the parasite as well as the associated trade-offs between traits and constraints on their evolution. Indeed, parasites harm their hosts in a number of ways that may be adaptive or not [2]. One source of negative effects of parasites on their hosts, adaptive to the parasites themselves, is the consumption of host resources by parasite growth and reproduction. Because host resources that are diverted by parasites are no longer available to the host, this must lead to a reduction in host fitness, termed virulence. If parasites that reproduce or transmit more do so by appropriating more host resources they should have stronger negative effects on their host, leading to a positive relationship between a parasite's transmission and its virulence, as is both predicted [2-5] and observed [[6], but see [7]]. From the point of view of the host, this would be expressed as a negative relationship between parasite transmission and host fitness [8,9]. Examining this relationship, *i.e*. how host fitness varies with parasite transmission success, has certain advantages because the different measures of virulence among studies are not always easily comparable nor do they necessarily imply the same thing for host or parasite fitness (*e.g*., host mortality versus weight loss).

When increased parasite virulence results in increased parasite transmission, modifying transmission may influence the evolutionary trajectory of virulence. This forms the theoretical basis for virulence management that proposes to modify virulence by altering transmission [10,11], though its practical applicability for disease management in natural systems is still hotly debated [6,12,13]. Indeed, some selection experiments have failed to observe an evolution of virulence in the predicted direction [14,15] even when using a single host genotype, thereby avoiding the complications due to the specificity of the infection phenotype among specific host and parasite combinations [16]. To date, environmental and/or host genetic variation are rarely taken into account when quantitative parasite traits are investigated though virulence and transmission ability are not traits of the parasite in isolation, depending on the host and environment it encounters [17-20], as has been commonly accepted for infection ability in plant-pathogen systems [21,22] or other host-parasite associations [23-25]. Therefore, to understand how parasite transmission affects host fitness we need to examine this relationship across a range of parasite and host genotypic combinations to better assess the role of each player in the host-parasite game and the nature of their shared phenotypic traits.

Here we examine the relationship between parasite and host fitness using all compatible combinations between seven parasite strains of the specialist oomycete, *Hyaloperonospora arabidopsis *(= *parasitica*) and six lines of its natural host *Arabidopsis thaliana *(Brassicaceae). With this experimental procedure we avoid as much as possible the variation due to gene-for-gene interactions for the qualitative compatibility between host and parasite genotypes, in order to concentrate on variation for quantitative phenotypic traits. *H. arabidopsis *is not lethal for its host, which enables us to measure both host and parasite fitness as the production of propagules, respectively seeds and asexual conidiospores. Furthermore, the fitness of an uninfected host gives a benchmark measure for how a healthy host would expend available resources on seed production and we test whether parasites that have increasing success in transmission are diverting increasing amounts of resources away from this primary goal for the host, both as a general tendency and in a specific way for different host or parasite genotypes.

## Results

Of the 210 inoculated plants 86.67% showed disease symptoms. No plants from the host line Fin showed disease symptoms when inoculated with Emco spores. One test plant each in ten of the 216 transmission tests using uninoculated control plants became infected eight days later. These 4.63% of cases represent either contaminations between test plants in the same container or errors in notation. This gives us an average total transmission of 0.46 target leaves for all controls plants, which can be considered as the error of measure for this variable.

### Transmission and Infection intensity

**Figure 1:**
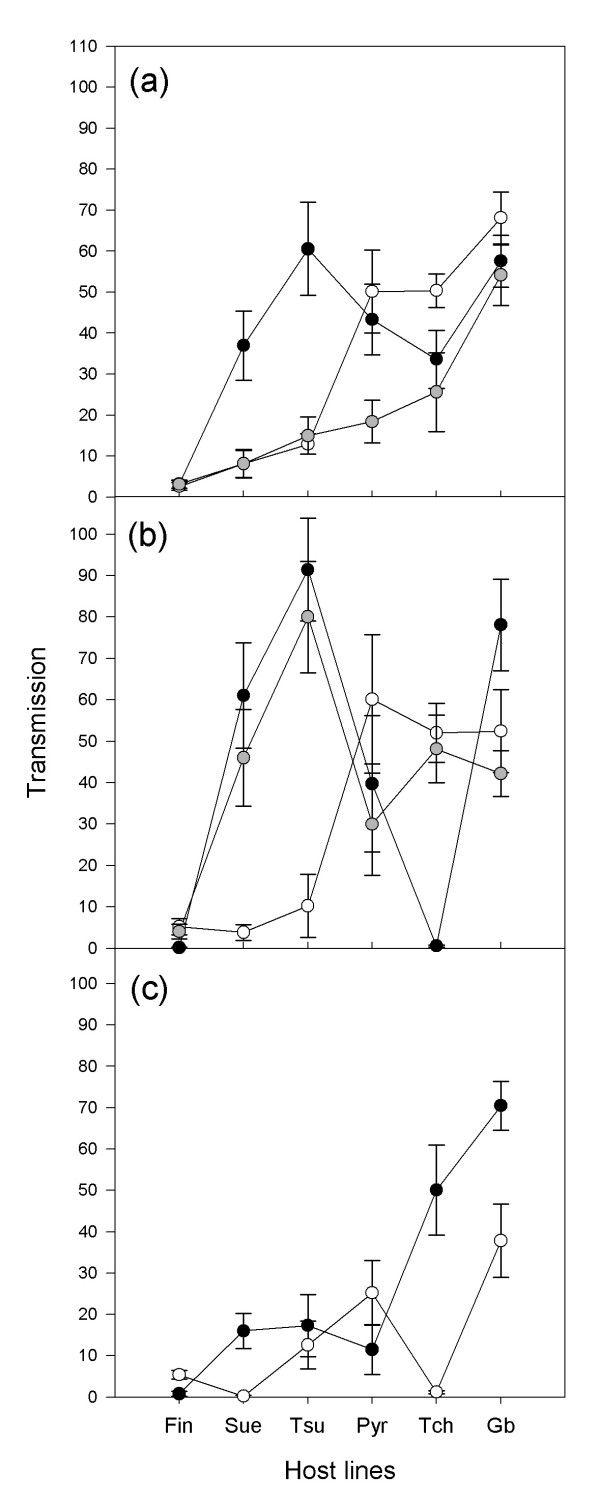
**Reaction norms of parasite transmission across the different host lines**. Parasite transmission (± SE) measured as the total number of successful transmissions. (a) Parasite strains averaged by origin: Laboratory (black dots), Fribourg (white dots) and Orsay (grey dots). (b) The three parasite strains of laboratory origin: Emco (black dots), Emwa (white dots) and Noco (grey dots). (c) The two parasite strains from Orsay: Ors 3 (black dots) and Ors 5 (white dots). Raw data are presented in the Figures though analyses were performed on square-root transformed data.

Transmission, estimated as the asymptote of the sigmoid curve fitted to the cumulated daily transmission data of the eight transmission events, differed among the different origins of the parasite strains, with the Orsay strains being the least transmitted, and among the six host lines. Transmission was highest on the host line Gb, followed by an indistinguishable group formed by Pyr, Tch and Tsu, followed by Sue and lowest transmission was on Fin (see Figure 1a). The interaction between host lines and origin of parasite strains was highly significant (Table 1). Indeed on the host lines Gb and Pyr the transmission was similar among the three origins whereas on Sue and Tsu the laboratory strains transmitted better than did wild strains. Furthermore, on the lines Pyr and Tch the strains from Fribourg transmitted more than those from Orsay (Figure 1a).

**Table 1:** Analyses of variance on the number of infected leaves and transmission

Source		Number of Infected Leaves			Transmission (Square root transformed)		
			
	Df	Type III SS	F		Type III SS	F	
Origin	2	47.64	7.95	**	100.63	15.53	***
Parasite [Origin]	4	66.28	5.53	**	56.24	4.34	*
Host	5	211.09	14.09	***	774.52	47.80	***
Origin × Host	10	72.11	2.41	*	198.23	6.12	***
Parasite × Host [Origin]	20	302.88	5.06	***	542.30	8.37	***
Error	168	503.20	-		544.42	-	

For transmission, the analyses are performed using a square root transformation.

* P < 0.05; ** P < 0.001; *** P < 0.0001

Transmission varied significantly by parasite strain, host line and their interaction even within some origins (Tables 1 and 2). Host line effect was always significant, the two parasite strains from Orsay differed significantly from each other, and significant interactions were found for Orsay and laboratory strains (Figure 1b and 1c).

**Table 2:** Analyses of variance within origins on the number of infected leaves and transmission

Origin	Source		Number of Infected Leaves			Transmission (Square root transformed)		
				
		Df	Type III SS	F		Type III SS	F	
Laboratory	Host	5	139.73	8.62	***	342.87	18.64	***
	Parasite	2	19.40	2.99	ns	22.47	3.05	ns
	Host × Parasite	10	249.27	7.70	***	419.95	11.42	***
	Error	72	233.20	-		264.88	-	
								
Fribourg	Host	5	85.73	5.13	**	424.40	25.50	***
	Parasite	1	0.07	0.02	ns	0.86	0.26	ns
	Host × Parasite	5	2.13	0.13	ns	2.99	0.18	ns
	Error	48	160.40	-		159.82	-	
								
Orsay	Host	5	68.28	5.98	**	202.04	16.20	***
	Parasite	1	46.82	20.50	***	32.92	13.20	**
	Host × Parasite	5	51.48	4.51	*	119.37	9.57	***
	Error	48	109.60	-		119.72	-	

For transmission, the analyses are performed using a square root transformation.

* P < 0.05; ** P < 0.001; *** P < 0.0001

The results were all qualitatively similar for the infection intensity on inoculated plants, measured as the number of infected leaves (Tables 1 and 2). All main and interaction effects remained significant when the host Fin, which was fully resistant to the parasite strain Emco, was excluded from the analyses.

### Relation between host and parasite fitness

**Figure 2:**
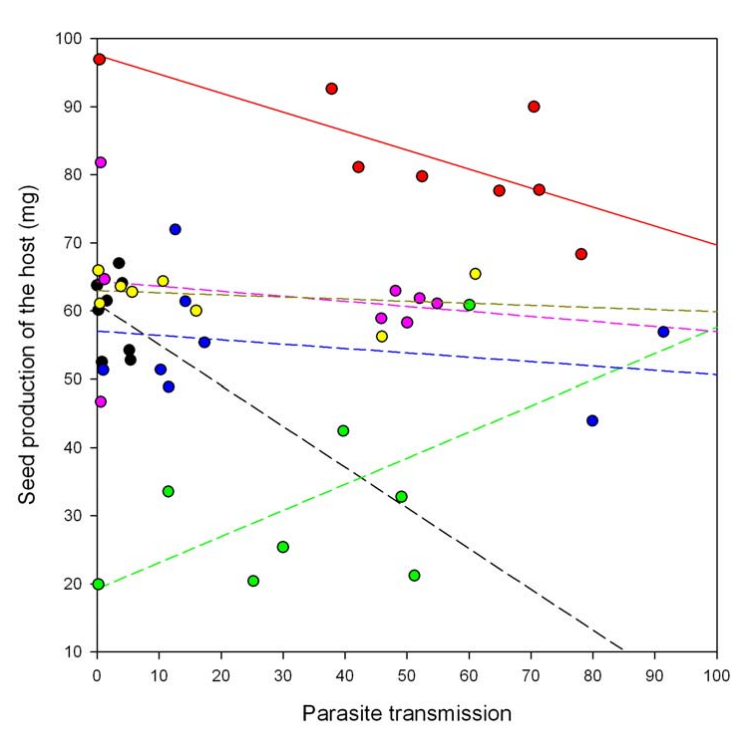
**Genetic correlations between host and parasite fitness**. Genetic correlations between host seed production (total mass of seeds in milligrams) and parasite transmission (number of successful transmissions). Each symbol represents a unique combination of host line and parasite strain. Symbols are distinguished according to the host line involved in the combination: Gb (red dots), Fin (black dots), Pyr (green dots), Sue (yellow dots), Tch (pink dots), Tsu (blue dots). Solid line: significant regression across the seven strains and control in combination with the host line Gb (*y *= -0.28*x *+ 97.58; Spearman'rho = 0.81, p-value = 0.015). Dashed lines: non significant regressions for the host lines Fin (*y *= -0.66*x *+ 61.32; ρ = -0.26, p-value = 0.53), Pyr (*y *= 0.43*x *+ 17.10; ρ = 0.55, p-value = 0.16), Sue (*y *= -0.03*x *+ 62.97; ρ = -0.29, p-value = 0.49), Tch (*y *= -0.07*x *+ 64.15; ρ = -0.24, p-value = 0.57), and Tsu (*y *= -0.06*x *+ 57.04; ρ = 0.17, p-value = 0.69).

Different host lines had different fecundities across the 48 combinations, including controls. We found no general genetic correlation between host fitness (seed production) and parasite fitness (transmission) but the interaction between host line and this covariable was significant, revealing that the slope of this relationship was not homogeneous (Table 3). Rank correlations calculated separately for each host line across the eight parasite treatments (seven parasite strains and one control) detected a significantly negative genetic correlation only for the host line Gb (Spearman's rho = -0.81; P-value = 0.015) (Figure 2).

**Table 3:** Analysis of covariance on the average seed production (in milligrams) by combination

Source	Df	Type III SS	F	
Host	5	9832.58	26.39	***
Parasite treatment	3	164.33	0.73	ns
Transmission	1	37.27	0.50	ns
Host × Transmission	5	1102.26	2.96	*
Parasite treatment × Transmission	3	225.39	1.01	ns
Error	30	2235.71		

* P < 0.05; *** P < 0.0001; ns: non significant

## Discussion

### Host genotype by parasite genotype interactions

Who controls the epidemiological traits of a host-parasite association is a key factor in predicting the evolution of this association [16,26]. Until recently, most models of host-parasite coevolution assumed that traits like symptom severity, transmission or virulence were the characteristics of the parasite only. Now an increasing body of evidence on plant-pathogen systems [20,27], microorganisms [19], invertebrates [17,28], and vertebrates [29] as hosts shows that both host and parasite genotypes may interact in the determination of the level of these quantitative traits. In our experiment we found that the intensity of infection (number of infected leaves) and the associated transmission of *H. arabidopsis *were influenced by strong interaction effects, with nonetheless significant main effects of parasite type on the one hand and host line on the other. The host line always explained a major part of the variance in transmission or infection intensity, with more differences among lines for the transmission (four non overlapping groups) than for infection intensity (three overlapping groups). This impact of host genotype on parasite fitness traits had already been demonstrated in the same association [20] but also in malaria models [29,30]. Contrary to theses precedent results [20] however, we demonstrate here, with a larger number of parasite strains, that the parasite also has a significant effect on transmission and infection intensity. Indeed, parasite success differed according to their origin, with isolates from Orsay succeeding on average significantly less well than Laboratory isolates. The two strains from Orsay, though they had been isolated from infected plants growing only a few meters apart, also differed significantly from one to another.

Some host lines suffered more infection than others and some parasite strains had undeniably better average infection success than others. These average results, however, hide large differences among specific combinations, as even "successful" parasite genotypes failed in particular combinations. Both host and parasite identities thus determined parasite fitness traits. Because increased performance on, or adaptation to, a particular host or parasite type will not necessarily imply increased performance in interaction with another host or parasite type, the selective landscape experienced by the two protagonists is unstable. Each new association of genotypes could "erase" any adaptation achieved with a previous partner preventing the appearance of a universally high-performance generalist. Clearly these genotype by genotype interactions will permit the maintenance of genetic variation for characters under selection as can genotype by environment interactions. This is particularly relevant in our pathosystem, as we found such variation in quantitative fitness traits within one natural population. Indeed, the two parasite strains Ors3 and Ors5, collected in the same host population in Orsay, showed significant interactions for infection phenotypes over the range of host lines tested. Though such significant genotype by genotype interactions within populations have been demonstrated for qualitative traits such as infectivity/susceptibility [23,24] there are very few demonstrations for quantitative traits [19,31]. Here we generated new, probably not previously encountered combinations of host and parasite because the six hosts originated from different geographic areas from each other and from the parasite strains we used. Despite the large geographical scale, however, these novel combinations may not be different in kind to those that occur naturally. *A. thaliana*, though a selfing plant, is highly variable for neutral markers, with much intra- as well as inter-population variation, the latter showing little geographical pattern [32-35]. Therefore a given parasite isolate might be regularly confronted with novel host genotypes, from a nearby population or even from the same population, that differ in qualitative and quantitative resistance.

### Trade-off between host and parasite fitness

The perfect organism should produce an infinite number of descendants immediately after its own birth. Indeed, following natural selection's rules, the best rate of reproduction should be strongly selected. So why are we not surrounded by such ideal organisms? A classical response is that reproduction is traded off against other traits that are also necessary for fitness. As an example, the number of descendants can be negatively correlated with their size or quality. In general, two traits are traded off if an increase in one leads to a decrease in the other because both require a common limited resource [36]. Though such trade-offs are logical and compelling, it has proven difficult to find evidence for them in natural systems. Indeed, the comparison of different allocation strategies should be made for individuals possessing the same amount of available resources [37], which is not often the case in natural systems and even hard to achieve in controlled experiments.

By analogy, parasites are considered to harm their hosts because they divert and consume a common resource that the host also requires for its maintenance and reproduction. Virulence is then the by-product of the parasite using its host's resources for parasite reproduction [2]. As a consequence, we expect that host resources are traded off between host and parasite fitness. Because of the implications for virulence evolution and management, the relationship between virulence (or traits linked to host fitness) and transmission (or parasite propagule production) has been investigated in many theoretical and experimental studies [see [6,38] for a review]. Several experimental studies have indeed shown a positive correlation between virulence and parasite fitness (or a negative correlation between host and parasite fitness) but the question of the relevance of such measures, and their applicability in the real world, remains [6]. Different kinds of approaches have been used to demonstrate this relationship. In general, they compare parasite isolates of varying degree of virulence on a single host line. These isolates could be either different parasite species of the same clade [39], different experimental treatments [40,41], different genotypes of the same parasite species [42-44] or different lines evolved under experimental selection [9,14,15,45-47].

Here in addition to using different parasite types we also used a number of host lines. This allowed us not only to test the relationship between host and parasite fitness but also to examine whether the nature of this relationship varied with host as well as with parasite identity. Globally, confounding all combinations, we found no correlation between host and parasite fitness [but see [8,20,29]]. However, we found significant heterogeneity for the relationship between host and parasite fitness among host lines, with a significant negative correlation for one of six, the line Gb. Interestingly, the Gb line also happened to be the most susceptible to *H. arabidopsis*, *i.e*. had the highest average transmission. In corollary we assessed how the relationship varied for each parasite origin exposed to a range of host lines. The slopes of the relationship between host and parasite fitness did not differ among the diverse parasite origins, although we had previously found, using a slightly different measure of host fitness, variation between two laboratory parasite strains [20]. This and our previous experiment [20] give inconsistent patterns for some combinations of host and parasite that were used in both experiments. For example the Noco strain transmitted on the Pyr ecotype in this experiment but had failed to do so in our previous experiment. However, these inconsistencies may be due to environmental differences between the two experimental conditions, as similar influences of environmental variation on infection phenotype are known [18].

**Figure 3:**
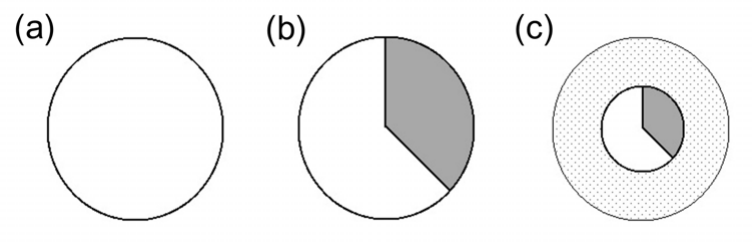
**Graphical model representing how resources allocated to reproduction between a host and its parasite may vary**. The surface of the circle represents the absolute amount of available resources accumulated by the host. The white portion is the amount of resources used by the host to produce its descendants, whereas the gray portion is diverted by the parasite to produce its own descendants. (a) non infected host: all the resources accumulated by the host are used for its reproduction. (b) host infected with a fully compatible parasite: an arbitrary proportion (a third in this case) of the resources accumulated by the host is diverted toward parasite reproduction. (c) host infected with a parasite with partially incompatible interaction: a part of the resources accumulated by the host are lost (dotted part of the circle), for instance due to the activation of host defenses, or to the inefficiency of resources use by the parasite. (c) illustrates the particular case where the relative diversion of resources by the parasite is the same as in case (b), but the absolute reproduction of both protagonists is lower.

**Figure 4:**
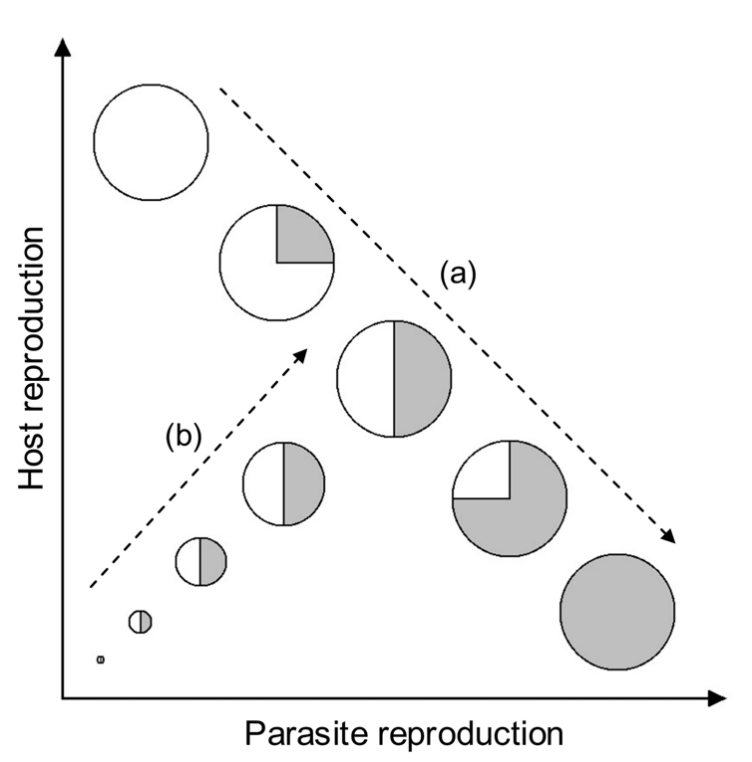
**Schematic representation of possible relationships between host and parasite fitness with the type of interaction described in figure 3**. In fully compatible combinations of host and parasite genotypes there is no loss of resources and the absolute amount of available resources is always maximal. In such a situation, as the proportion diverted by the parasite increases, the portion left to the host, and thus its reproduction, decreases (relationship (a)). However, if all combinations are not fully compatible, host and parasite reproduction will also depend on the level of compatibility, *i.e*. the amount of lost resources. In this latter case, an increase in compatibility will increase both host and parasite reproduction and lead to a positive relationship between host and parasite fitness (relationship (b)).

Why did we observe a negative relationship between host and parasite reproductive success so rarely? Clearly, if hosts and parasites use the same resource base for reproduction there must be a trade-off between their respective fitness. However, we succeeded in revealing this in only one case. One possibility is that the asexual transmission success of the parasite that we measured in this experiment is not a good estimator of the global fitness via both asexual and sexual stages. Of course, the production of sexual oospores also consumes some host resources, and an additional trade-off between these two modes of transmissions could explain why, in some cases, infections with poor asexual transmission greatly reduced host fitness (and *vice versa*). However different parasite strategies for allocating host-derived resources to sexual versus asexual reproduction does not explain our observation that the relationship between host fitness and parasite asexual transmission differed among host lines, unless this potential trade-off also depended on host genotype by parasite genotype interactions. We thus propose a more general hypothesis consistent with our results. Superimposed upon underlying variation in host size and resource availability, that were standardized as much as possible in our experiment, shared host and parasite control of the infection phenotype may directly modify this resource pool. As we have discussed above, each combination represents a particular specificity between host and parasite. This specificity could, in addition to influencing the infection phenotype, control the amount of resources available that can be converted into both host and parasite (asexual or sexual) reproduction, thus blurring any trade off for the allocation of these resources. We imagine that particularly compatible interactions reduce the shared resource pool little while conflictual ones leave little resource for either host or parasite. By "compatibility" we mean, here, the adequacy of the host-parasite association from a quantitative point of view, rather than the qualitative ability of the parasite to infect which is how this term is employed in the gene for gene literature. As an example, activating defense systems in plants [48], insects [49], and vertebrates [50] is costly so parasites that induce or hosts that mount a strong defensive reaction will reduce the total resource pool available for reproduction of both parties. In addition incompatible parasites may be less efficient in resources conversion or may consume "dead end" tissues that do not permit their dissemination [2]. We propose that different interactions vary both for their compatibility, hence total resource pool available for host and parasite reproduction, and for the proportion of resources appropriated for parasite reproduction alone (Figure 3 gives a representation of hypothetical partitioning of resources toward host and parasite reproduction). When only this proportion varies, negative correlations are expected between host and parasite reproduction. When only compatibility varies, positive relationships should be found because increasing compatibility would then increase the resource pool available for the parasite but also the residual resources available for the host (hypothetical relationships are represented in Figure 4). Variation in both could generate the large number of possible relationships between host and parasite reproductive success that we observe (Figure 2). Under this hypothesis we propose that negative relationships would be found more often in systems with highly susceptible hosts, which are uniformly compatible to all parasite genotypes, such as the host line Gb in our experiment. The systematic use of highly susceptible hosts in host-parasite studies would then hide the diversity of possible relationship between traits such as parasite and host fitness.

## Conclusion

The phenotype of a host-parasite association is under the joint control of both protagonists. We have seen that these genotype by genotype interactions can lead to profound differences in the quantitative traits expressed during infection when different combinations are considered. More importantly, these interactions are still present at the local scale, between parasite strains from the same population. We argue that if new combinations of genotypes are frequent, then host and parasite may be unable to achieve any global adaptation of one to the other. These interactions should thus be considered as another level of heterogeneity, similar to environmental heterogeneity, that affects host-parasite coevolution.

The shared control of phenotypic traits of the infection, including host and parasite fitness, may also influence the relationship between these traits. Indeed, even if, in theory, host and parasite fitness are traded off against each other, as for any two functions that use a common resource, variation in the compatibility of particular parasites to particular hosts may render the relationship between host and parasite fitness unpredictable. Increased parasite transmission does not necessarily come at an increased cost to all hosts. As a consequence, virulence evolution may not always be manageable by modifying transmission success except in systems with little variation in basic compatibility or little host genetic diversity. Virulence management of human diseases, which has aroused much interest but remains controversial, might not be globally applicable.

## Methods

### Materials

The oomycete *Hyaloperonospora arabidopsis *(= *parasitica*) is a natural pathogen of the Brassicaceae *Arabidopsis thaliana*, its specific host. This biotrophic parasite causes the loss of some rosette leaves, but only exceptionally kills host plants when they are infected after the true leaves have appeared. The major symptom of the infection is the production of conidiophores on the surface of leaves a few days after infection, giving this parasite its name "downy mildew". These conidiophores bear packets of spores that are the asexual stage of *H. arabidopsis *and can be transmitted to other plants. The parasite also reproduces sexually via oospores that remain within leaves until host death, and then can reinfect seedlings the next season. [51]. Reproduction by oospores is critical for the survival of this pathogen between the active growing seasons of its host but plays no role in within-season dynamics. Asexual reproduction via conidiospores, on the other hand, is responsible for dissemination and epidemic dynamics within populations and seasons. Therefore we concentrated on only the asexual stage of this pathogen. The seven strains used in the experiments were of three different origins. Three of them, Emco, Emwa and Noco, were "laboratory strains" originated from isolates collected more than ten years ago and since maintained artificially as asexual cultures on specific *A. thaliana *lines [52]. They were provided by the Sainsbury Laboratory (John Innes Center, Norwich, U.K.). A second type of strains, Ors 3 and Ors 5, were collected in spring 2004 from conidiospores on infected host plants of the same population on the campus of Université Paris-Sud, Orsay, France. The last strains, Fri 3 and Fri 5, were obtained from oospores of two infected plants also sampled in spring 2004 in a population in Fribourg, Switzerland. In both Fribourg and Orsay populations the sampled plants were situated a few meters apart. For Fribourg strains seedlings were experimentally infected with each natural oospore isolate and a single infected seedling per isolate was retained as spore source for subsequent infections. Conidiospores were multiplied for 22 asexual generations for the Orsay strains and 6–7 asexual generations for the Fribourg strains before the experiment. Thus they are likely to represent only a single genotype per strain. These wild strains were tested for infectivity profiles on a series of host lines. All strains, except the two Fribourg ones were distinguishable from each other.

Of the six *A. thaliana *lines used as hosts in the experiments, five were generated from at least two generations of selfing of plants issued from seed collected in wild populations across Europe: Finland [Fin], England [Gb], Pyrenees [Pyr], Sweden [Sue], and Czech Republic [Tch]. The sixth line was the registered ecotype Tsu, originally sampled in Japan. Our purpose was to test a maximum number of parasite and host strains in a complete matrix of interactions. Major genes for resistance against this parasite are, however, common [51] and it was difficult to find a large range of ecotypes susceptible to all parasites. Therefore we included a host ecotype (Fin) that was resistant to Emco but susceptible to the other six strains. All other hosts were susceptible to all seven parasite strains.

### Controlled cross inoculation experiment

Every host line was subjected to eight treatments, an inoculation with a spore suspension of each of the seven *H. arabidopsis *strains or a mock inoculation with pure water (control treatment). Five replicates were carried out for each of these 48 combinations. The seeds of each host lines were sown the same day in 5 × 5 × 5 cm compost pots then randomized and placed in the dark at 5–6°C ten days in order to synchronize germination. Seedlings were then grown up to 4–6 leaves in the greenhouse with natural photoperiod (23°C day – 15°C night). Supernumerary seedlings were removed after germination in order to keep only the most central plant in each pot. After 18 days in the greenhouse all the plants receiving the same parasite treatment were regrouped and inoculated with a 8 μL drop of spore suspension for the two largest leaves and a 4 μL drop on all smaller leaves of the corresponding parasite strain, or of pure water for the control treatment. The seven spore suspensions were diluted with water to 6 × 10^4 ^spores per mL [53]. Each plant was then placed in its own closed transparent plastic cylinder to prevent contamination and maintain high hygrometry, and then randomly arranged in a growth chamber (10:14 light-dark photoperiod, 16°C ± 3°C average temperature and hygrometry around 95–100%). From the seventh day after inoculation the plants were individually checked twice a week during four weeks, which corresponds to the usual duration of the symptoms in such conditions. At each observation the number of infected leaves and their position on the plant was recorded, and the plant was allowed to transmit spores to a group of three new healthy seedlings at the 4–6 leaves stage of the same host line ("test plants"), following the protocol described in [20]. All the pots containing the test plants inoculated during a same transmission event were kept together in six trays with plastic covers in the same growth chamber as the observed plants. The daily transmission success was then estimated by counting the number of infected leaves on the three test plants eight days after the transmission event. The total transmission success ("transmission") over the whole infection period was estimated as the asymptote of the sigmoid curve fitted to the cumulated daily transmission data of the eight transmission events. When zero or only one out of the eight transmission events led to a successful infection of the test plants, the variable transmission was arbitrary given the value of the cumulative transmission at the eighth day. The total number of infected leaves of each plant was estimated by summing all newly infected leaves over the eight observations. After 35 days in the growth chamber the plants were moved again into the greenhouse (natural photoperiod 23°C day – 15°C night) to complete their life cycle. At that time the covers of the plastic cylinders were removed in order to lower the hygrometry. Plants were watered *ad libitum *until flowering and then harvested regularly as fruits matured, in order to collect all the seeds before they fell from open fruits. The total weight of all the seeds produced by each plant (measured to the precision of 1/1000 g) was used as our estimate for host fitness. Because *A. thaliana *is annual, this variable represents its lifespan investment in reproduction.

### Statistical analyses

As there were doubts about a possible contamination of three plants belonging to the control treatments of the respectively Fin, Pyr and Tch host lines, these plants were not included in the statistical analyses. Statistical analyses were performed with JMP version 5.1.2 (SAS institute, Cary, NC). We used nested analyses of variances (ANOVA) to examine the effects of Origin (Laboratory strains, Fribourg and Orsay), Parasite strain (nested within Origin), Host line and the interactions between Host line and Origin, and Host line and Parasite strain on transmission and the number of infected leaves of all the inoculated treatments. ANOVAs were then performed independently for each of the three different origins to test for Host line, Parasite line and Interaction effects on the same variables. As the distribution of the variable transmission was very asymmetrical due to a large number of zeros this variable was subsequently modified with a square root transformation ((Transmission)^0.5^) in all the ANOVA analyses to obtain residuals as close as possible to a normal distribution. An ANCOVA was carried out to test the relationship between host and parasite fitness for each specific host and parasite combination using mean seed production and transmission of each of the 48 combinations, including the controls. We chose to analyze seed production of control and infected combinations itself instead of a measure of virulence estimated from the difference between healthy and infected plants because this maximizes the information used and permits each host line its own starting point specific to its fecundity. In this analysis, the "Parasite treatment" variable included the three parasite types (Laboratory, Fribourg and Orsay) and Controls. We tested the Host line and Parasite treatment as main effects (the parasite line effect nested within Parasite treatment was removed as it explained almost none of the variance in the model), transmission as covariable and the effects of the interaction term between this covariable and the two main effects. This model enabled us to compare the slopes of the relationship for the different host lines (Host line × Transmission term) and the different parasite origins (Parasite treatment × Transmission term). Note that in these interaction terms the covariable was centered at zero by subtracting its mean so that means rather than intercepts were compared for the main effects.

## Authors' contributions

LS carried out the experimental work, performed the statistical analyses and drafted the manuscript. VH participated in the design of the study and assisted the experimental work. JAS contributed to the experimental design, data analysis and writing. All authors read and approved the final manuscript.
